# From Formulation to Application: Effects of Plasticizer on the Printability of Fluoro Elastomer Compounds and Additive Manufacturing of Specialized Seals

**DOI:** 10.3390/mi15050622

**Published:** 2024-05-05

**Authors:** Mookkan Periyasamy, AA Mubasshir, Stiven Kodra, Sangeetham Chandramouli, Ronald Campbell, David O. Kazmer, Joey L. Mead

**Affiliations:** 1 Greene Tweed & Company LLC, 1684 South Broad St, P.O. Box 1307, Lansdale, PA 19946, USA; rcampbell@gtweed.com; 2 Department of Plastics Engineering, University of Massachusetts, Lowell, MA 01854, USA; aa_mubasshir@uml.edu (A.M.); david_kazmer@uml.edu (D.O.K.); joey_mead@uml.edu (J.L.M.); 3 Department of Mechanical Engineering, University of Massachusetts, Lowell, MA 01854, USA; stiven_kodra@uml.edu (S.K.); chandramouli_sangeetham@student.uml.edu (S.C.)

**Keywords:** additive ram material extruder (ARME), additive manufacturing, thermoset elastomers, voids and shrinkage, support for 3D printing, soft tooling, mechanical characterization, plasticizer in 3D-printed elastomers, additive manufacturing of seals

## Abstract

This work investigated material extrusion additive manufacturing (MatEx AM) of specialized fluoroelastomer (FKM) compounds for applications in rubber seals and gaskets. The influence of a commercially available perfluoropolyether (PFPE) plasticizer on the printability of a control FKM rubber compound was studied using a custom-designed ram material extruder, Additive Ram Material Extruder (ARME), for printing fully compounded thermoset elastomers. The plasticizer’s effectiveness was assessed based on its ability to address challenges such as high compound viscosity and post-print shrinkage, as well as its impact on interlayer adhesion. The addition of the PFPE plasticizer significantly reduced the FKM compound’s viscosity (by 70%) and post-print shrinkage (by 65%). While the addition of the plasticizer decreased the tensile strength of the control compound, specimens printed with the plasticized FKM retained 34% of the tensile strength of compression-molded samples, compared to only 23% for the unplasticized compound. Finally, the feasibility of seals and gaskets manufacturing using both conventional and unconventional additive manufacturing (AM) approaches was explored. A hybrid method combining AM and soft tooling for compression molding emerged as the optimal method for seal and gasket fabrication.

## 1. Introduction

Among the vulcanized rubbers used for sealing and gasket applications, fluoro-elastomers (FKM) and perfluoro-elastomers (FFKM) are preferred because of their excellent heat and oil resistance [1]. The continuous drive for energy efficiency and reduced emission is continuously pushing the operating temperature of power-generating instruments upwards, and, in many cases, the end-use temperature for seals exceeds 200 °C. At such elevated temperatures, only FKMs and FFKMs provide meaningful sealing performance [2]. At lower temperatures, FKMs and FFKMs also remain highly relevant because of their broad chemical resistance [2]. The excellent properties of FKMs and FFKMs come from the C-F bond in their polymeric chains, which is the strongest bond in chemistry [3]. 

The predominant manufacturing techniques for FKMs and FFKMs are compression molding, injection molding, and transfer molding. As with any manufacturing industry dependent on molding processes for product manufacturing, a considerable gap in the timeframe exists between the product idea conceptualization and mass manufacturing of products. Detailed design, prototyping, and testing are required to finalize the product design and specification before mass manufacturing starts [4]. As the rubber industry is heavily dependent on molding processes, the intermediate stages between conceptualization and mass production require considerable time and resources. In this context, additive manufacturing (AM) emerges as a crucial bridge between product conceptualization and mass production, dramatically reducing both costs and lead times. By minimizing or eliminating the need for tooling molds and dies during the prototyping phase, AM accelerates the process. Additionally, it facilitates the swift and cost-effective creation of prototypes for iterative design improvements and rigorous testing. This study is an attempt to combine the excellent properties of FKMs with the design freedom and rapid prototyping attributes of AM to open up new application possibilities.

From the shoes beneath our feet to the tires meeting the road, from waterproofing gaskets for our homes to the precision seals of ultra-clean semiconductor chip processing chambers, vulcanized rubber is one such material class that seamlessly integrates into the fabric of our daily lives [5]. Key prominent vulcanizable rubbers are natural rubber (NR), nitrile butadiene rubber (NBR), styrene–butadiene rubber (SBR), ethylene propylene diene monomer rubber (EPDM), isobutylene isoprene rubber (IIR), isoprene rubber (IR), alkyl acrylate copolymer (ACM), ethylene acrylic elastomers (AEM), hydrogenated nitrile butadiene rubber (HNBR), and many others [6]. When these rubbers are mixed with specific additives and curatives to obtain desired properties, they are called fully compounded thermoset elastomer or rubber [5]. Although AM of different elastomeric materials using various methods has been discussed extensively in the literature [7,8,9,10], very few scientific articles can be found regarding AM of fully compounded thermoset elastomers or traditional rubber compounds. Recently, the literature reports have started to emerge demonstrating the fabrication of parts from several types of rubbers, such as NBR, HNBR, and silicones, using various AM processes [11,12,13,14,15,16,17]. However, we could find very little work in the literature discussing AM of FKM rubbers or FKM rubber compounds. Previously, we reported the feasibility of fabricating seals and parts from FKM and FFKM filaments via the fused filament fabrication (FFF) process [18]. One of the most critical challenges of printing FKM rubber compounds or any other traditional rubber compounds via the FFF process is the insufficient stiffness of the rubber filaments. This was solved in our previous work by in situ freezing the filament below its glass transition temperature using dry ice [18]. However, the FFF printing approach has demonstrated complications for manufacturing complex and specialized seals, gaskets, and other parts due to the narrow process latitude and reproducibility. In this paper, we report how the process latitude can be improved by employing a barrel-plunger material extrusion system.

Traditional rubbers are usually not used in the industry without some form of compounding to achieve the required end-use properties of the parts made from them. Compounding of rubbers usually involves mixing the rubber stock with various chemical additives such as accelerators, accelerator activators, anti-degradants, and processing aids; different reinforcing fillers such as carbon black, silica, clay, and glass fiber; and curing agents such as sulfur, organic peroxides, and metal oxides [5]. As a result, a rubber compound becomes a very high-viscosity material system. This elevated viscosity of traditional rubber compounds gives rise to critical challenges in fully compounded rubber AM, such as post-print shrinkage and limited time available for printing. In our previous work, a liquid NBR polymer was used as a viscosity modifier to fine-tune a traditional NBR compound for material extrusion additive manufacturing (MatEx AM) applications and to resolve the critical challenges associated with MatEx AM of rubbers [19]. The liquid NBR proved to be highly effective in resolving many of the challenges. In this study, we inspected the effectiveness of a plasticizer as a viscosity modifier and reported on its effect on the overall printability of an FKM compound.

Plasticizers are substances, usually liquids, added to polymers or polymer compounds to increase their flexibility, processability, and workability. They act as internal lubricants between polymer chains, reducing friction and making the polymer softer and easier to shape. In the rubber industry, plasticizers are usually used as a processing aid to reduce shear during mixing, improve lubrication during processing, prevent sticking to the mold surface, and provide additional stability by increasing the internal adhesion in emulsions. Plasticizers can also be used to lower the viscosity of the rubber compound [20]. The use of plasticizers in material development for AM applications is not uncommon. Bajwa et al. [21] evaluated the effects of three different types of plasticizers on the mechanical and thermal properties of polylactic acid (PLA) filaments for AM. They observed significant improvement in elongation at break and reduction in ultimate tensile strength. Oz et al. [22] reported the effects of polyethylene oxide (PEO) as a plasticizer on the mechanical and joint properties of carbon fiber powder (CFP)-reinforced PLA filaments for AM. Their work concluded that incorporating the PEO plasticizer improved printed parts’ ductility but reduced strength. Similarly, Menčík et al. [23] reported improved elongation at the break of printed parts using plasticizers in Poly(3-hydroxybutyrate)/Poly (lactic acid) filaments. Wasti et al. [24] evaluated the effects of two plasticizers (polyethylene glycol (PEG) 2000 and Struktol TR451) on the thermal and mechanical properties of bio-composite filaments made from lignin and polylactic acid for AM. They reported that the use of PEG as a plasticizer improved both tensile stress and elongation at break, whereas the use of Struktol TR451 as a plasticizer only improved the elongation at break. However, we could not find any information in the literature on the effect of plasticizers on the printability and mechanical behavior of additively manufactured parts from fully compounded FKM rubber compounds or any other thermoset elastomer compounds. This study employs the viscosity-modifying attribute of plasticizers to improve the printability of an FKM rubber compound. This article also intends to shed light on the effect of a commercial plasticizer on the mechanical properties of additively manufactured parts from fully compounded FKM rubber compounds.

The work presented in this article can be broadly divided into two sections. First, we evaluated the effectiveness of a plasticizer as a viscosity modifier and its effects on the printability of an FKM compound. In this regard, we compounded two FKM formulations. The first formulation, FKM-1, contained no plasticizer and acted as a control. The second formulation, FKM-2, had a commercial plasticizer for FKM rubber compounds. These two compounds were evaluated based on their viscosity, post-print shrinkage, tensile properties, and interlayer adhesion. Based on the performance of the FKM compounds on the parameters mentioned above, we drew a conclusion on their printability and reported in the first half of the result and discussion section (Section 3.1). Second, we evaluated the feasibility of rubber AM technology to fabricate FKM seals and gaskets for prototyping purposes. In this context, several conventional and unconventional AM approaches were evaluated and reported in the second half of the result and discussion section (Section 3.2).

## 2. Materials and Methods

### 2.1. Materials

The base fluoro-elastomer or FKM rubber stock was purchased as Tecnoflon VPL 45535 from Solvay (10 Leonard Ln, West Deptford, NJ, USA). FKM rubber cure package was obtained from R.T. Vanderbilt Company, Inc. (30 Winfield St, Norwalk, CT , USA), and silica filler was purchased from Fiberglass supply, Inc. (11824 water tank road, Burlington, WA , USA). The plasticizer, Fomblin M60, was purchased from Solvay (10 Leonard Ln, West Deptford, NJ , USA).

### 2.2. Preparation of Rubber Compounds

The formulations of the two FKM rubber compounds evaluated in this study are reported in Table 1. This study explores the printability of two custom FKM rubber formulations (FKM-1 and FKM-2) designed for applications such as seals and gaskets [25]. FKM 1 served as the control material. A 50 g batch of each compound was mixed using a Brabender Plasticorder Intelli-Torque Plus internal mixer (Model 01-55-000, Duisburg, Germany) with counter-rotating screws. The ingredients were incorporated into the mix following the order in Table 1. First, the rubber was kneaded for 2 min. Fillers were then added and mixed for an additional 5 min to ensure proper dispersion. Next came the curing agents, adhering to the quantities listed in Table 1. To investigate the influence of a plasticizer on additive manufacturing and product performance, 5 parts per hundred rubber (PHR) of the plasticizer was included only in the FKM-2 formulation. For both compounds, mixing continued after the final ingredient addition until the mixing torque stabilized, indicating complete mixing. The total mixing time for all compounds was 18 min.

### 2.3. Additive Manufacturing

**Figure 1:**
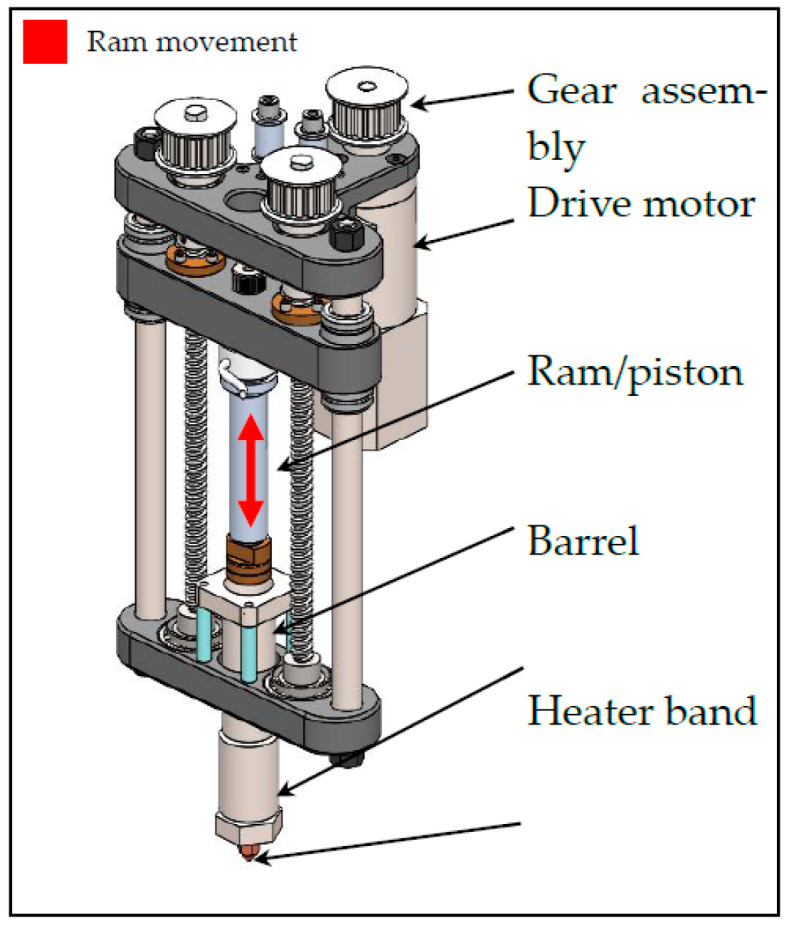
Three-dimensional model of the Additive Ram Material Extruder-3XL (ARME-3XL) printhead.

All 3D prints were conducted using a custom print head (shown in Figure 1) mounted on an Ender 5 pro with modified firmware. The printer (called ARME 3XL) shown in Figure 1 is an updated version of the Additive Ram Material Extruder (ARME) discussed in previous work [16], with a stronger stepper motor and lead screws on both sides of the barrel piston setup. Before printing, rubber compounds (formulated as outlined in Section 2.2) were loaded into the ARME 3XL barrel and heated under compression for a 5 min period. Then, the printing process of the desired geometry was started. All specimens were printed at 10 mm/sec print speed and 100 °C printing temperature. The bed was kept at room temperature. A nozzle with a 0.8 mm orifice diameter was used, and the layer height was kept constant at 0.4 mm for all prints. Completed prints were removed and transferred to an oven for curing. All specimens underwent a 15 min cure cycle at 160 °C.

### 2.4. Characterization of Rubber Compounds

#### 2.4.1. Cure Behavior and Formulation Viscosity

To analyze the rheological and cure properties of each rubber formulation, an oscillating disk cure meter (RPA 2000, Alpha Technology, Hudson, OH, USA) was used, following the guidelines of ASTM D2084 [26]. Moreover, 10 g uncured rubber samples were used as specimens to be tested with the RPA. Cure profiles were evaluated at both the print temperature (100 °C) and the designated cure temperature (160 °C). All compounds were tested with a constant frequency of 100 CPM and a rotational amplitude of 1°.

#### 2.4.2. Post-print Shrinkage

**Figure 2:**
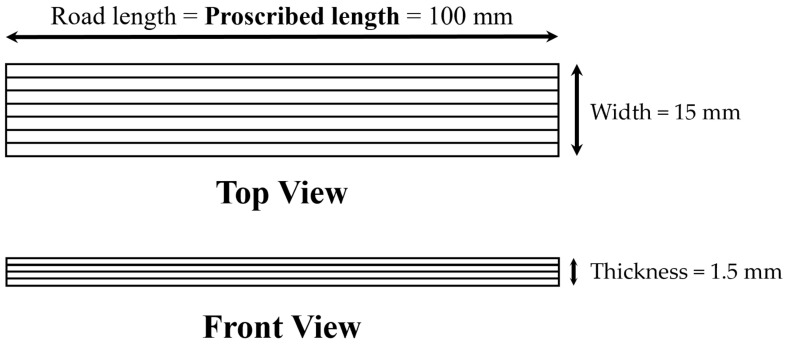
Schematic representation (not to scale) of specimen dimension and print pattern used for post-print shrinkage measurement.

Post-print shrinkage was measured using a standardized rectangular test part (100 mm long, 15 mm wide, 1.2 mm thick). The direction of print was kept aligned to the longest arm of the rectangle, and a regular serpentine print pattern was used. Figure 2 illustrates the dimensions and printing pattern of the part used for post-print shrinkage measurement. Five samples were printed for each FKM compound. Dimensions of printed rubber samples were recorded before and after the completion of their thermal cure. From the dimensions of the fully cured printed part, their post-print shrinkage was measured as the percentage of reduction in length from the original length (or proscribed length). 

#### 2.4.3. Tensile Behavior

**Figure 3:**
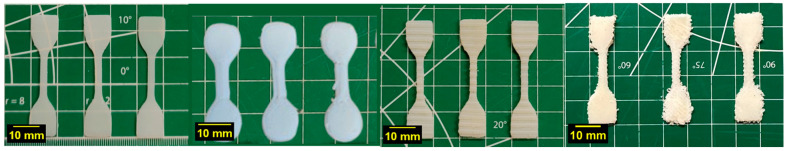
Compression-molded and three types of printed tensile specimens tested for each rubber compound. Sequentially from left to right: compression-molded, printed: parallel to applied force, printed: normal to applied force, and printed: zigzag (+45°/−45°). All tensile specimens were prepared following the ASTM D412—Type C tensile specimen at half-scale.

Tensile properties of printed specimens from the two rubber formulations were characterized. Three different types of printed specimens were evaluated in this study: printed (concentric) where the roads were parallel to the direction of applied force, printed (transverse) where the roads were normal to the direction of applied force, and printed (zigzag) where roads were at +45°/−45° angle with the direction of applied force. The tensile properties of printed specimens were compared against compression-molded specimens, which were die cut from a compression-molded sheet using the same die. Compression-molded sheets were formed using a 152 mm × 152 mm mold cavity with 1.5 mm thickness. To cure the elastomer compounds, the cavity was kept at a clamp force of 25 kN at 160 °C for 15 min. Tensile specimens were then die cut from the compression molded sheet using a half-scale ASTM D412-Type C die [27]. All tensile specimens were tested using a universal test machine (Instron 4466, Norwood, MA, USA) at a crosshead speed of 500 mm/min in accordance with ASTM D412. An extensometer (Instron 2603-086, Norwood, MA, USA) was used to record elongation of the specimens. Figure 3 shows the compression-molded, printed (axial), printed (transverse), and printed (zigzag) samples side by side.

## 3. Results and Discussion

### 3.1. Effect of Material Formulation

#### 3.1.1. Rheology and Cure Behavior of FKM Compounds

**Figure 4:**
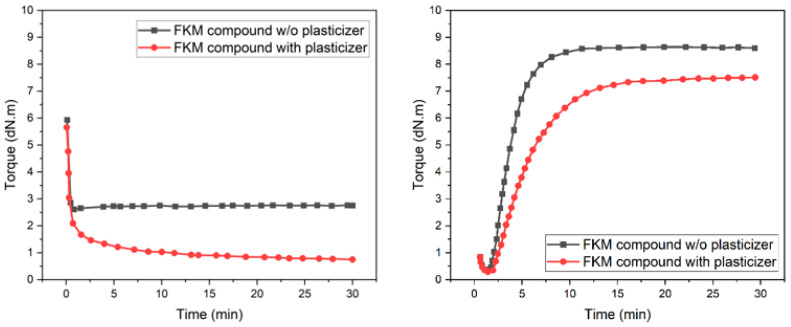
Cure curve of FKM compounds at printing temperature of 100 °C (**left**) and oven cure temperature of 160 °C (**right**).

The presence of crosslinks in thermoset elastomers is the principal difference between a thermoplastic and thermoset material. Vulcanizing agents present in a thermoset compound, such as sulfur, organic peroxides, and metal oxides, form crosslinks with polymeric chains of the elastomer when the rubber compound is exposed to an elevated temperature and, in turn, yield more robust and thermally stable elastomers. This process of crosslink formation is called curing [28,29,30]. Vulcanization, as a process, comprises three distinct phases: the induction phase, the curing phase, and lastly, the overcure phase [5]. Rubber Process Analyzers (RPAs), a type of oscillating disk cure meter, are used extensively in the rubber industry to track vulcanization progress. This is achieved by measuring the torque needed to deform a rubber sample over time, generating a ‘cure curve’ [31]. Within the industry, torque values are broadly correlated with viscosity [32]. For this discussion, subsequent references to FKM compound viscosities will be in relation to Figure 4a,b, with units expressed in dN.m.

When a rubber compound is exposed to an elevated temperature, the induction phase of vulcanization begins. As a result, crosslinks start to form, resulting in only a modest increase in the viscosity of the rubber stock. During this induction phase, the rubber stock remains processable and exhibits fluid-like behavior [31]. MatEx AM of the FKM rubber compound using the ARME 3XL printhead was conducted in this phase. Since one of the most critical factors affecting the printability of a fully compounded thermoset elastomer is its viscosity, it is essential to evaluate the compound’s viscosity during this stage at the print temperature. Figure 4a shows the viscosity of the FKM rubber compounds at the print temperature of 100 °C. 

It is evident from Figure 4a that the FKM-2 compound with plasticizer showed lower viscosity than the FKM-1 compound without plasticizer. This behavior was expected and can be attributed to the small molecular size of the plasticizer. These small plasticizer molecules are located in between the long polymeric chains, acting like lubricants and increasing the spacing between the chains, known as ‘free volume’. With more space, the polymer chains can slide past each other more easily, leading to less resistance to flow or lower viscosity [20]. It is also noticeable, from Figure 4a, that the viscosity of both compounds remained stable throughout the printing process, which lasted less than 30 min in all cases.

At the completion of the printing process, the printed parts were removed from the print bed and placed into a convection oven for curing at 160 °C. The cure curve is also used in the rubber industry to determine the optimum cure time for any compound at a specific temperature. The time required to reach 90% of maximum torque is considered the optimum cure time (t_90) [5,31,32]. It can be inferred from Figure 4b, showing the cure curve of both FKM compounds at the cure temperature of 160 °C, that the FKM compounds reach optimum cure around 15 min at 160 °C. 

Last, the maximum torque value of a compound in a cure curve is usually used as an indication of the rubber compound’s modulus in the rubber industry [32]. Figure 4b shows that the FKM compound with the plasticizer had a lower maximum torque value than the compound without the plasticizer. This suggests that the FKM compound with the plasticizer had a comparatively lower modulus than the FKM compound without the plasticizer. Again, such behavior was expected as the increased free volume contributed by the plasticizer would allow the polymer chains to move and slide past each other more easily, thus making the rubber compound more flexible and less deformation-resistant [20,33].

#### 3.1.2. Post-print Shrinkage

When a strand of fully compounded rubber is extruded through the printer nozzle and allowed to relax, it shrinks in size in the direction of extrusion. This phenomenon is defined as post-extrusion or post-print shrinkage. Post-print shrinkage was found in this research to be one of the most, if not the most, critical issues in MatEx AM of fully compounded rubbers or thermoset elastomers. This phenomenon can be attributed to the inherent characteristic of polymeric chains to revert to their initial state of relaxation (random coil chain) after they have been stretched [34,35,36]. During the printing process, the FKM compounds were extruded through the small opening of the printer nozzle. This process induced stresses on the polymeric chains and elongated the coiled chains. Once the extruded road was laid on the print bed, the induced stress slowly dissipated due to the viscoelastic nature of rubber compounds. As the induced stress dissipated, the polymeric chains of the extruded road returned to their coiled state from the elongated state. This behavior of the polymeric chains gives rise to the post-print shrinking phenomena in MatEx AM of rubbers. 

**Figure 5:**
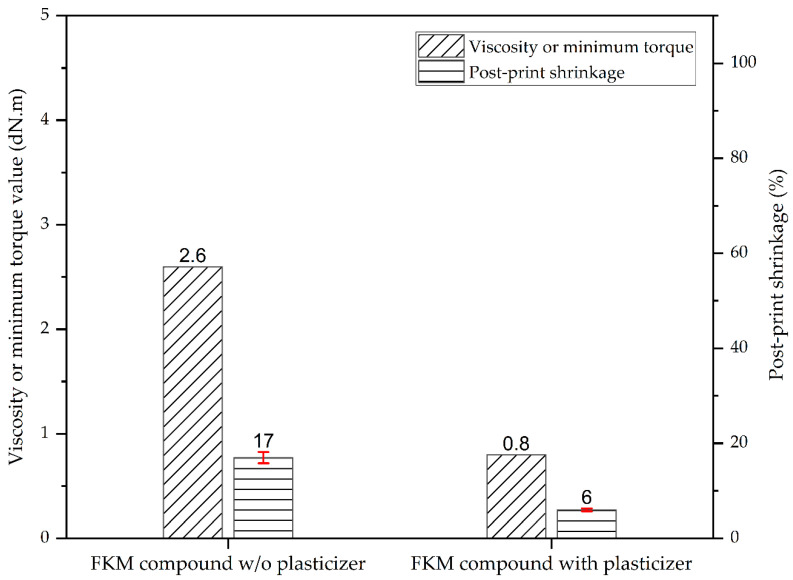
Correlation between the FKM compound viscosity and post-print shrinkage.

Post-print shrinkage is an important concern for rubber AM technology since this phenomenon results in dimensional inaccuracy. The severity of the dimensional inaccuracy depends on the degree of post-print shrinkage of the rubber compound used for printing. As a critical application of FKM rubbers are seals and gaskets for high-temperature and high-pressure applications for industries such as semi-conductor and oil drilling, achieving dimensional accuracy of the printed parts is very important to enable rubber AM technology to be a viable alternative manufacturing technique. In our previous work [19], we have demonstrated the efficacy of lowering the compound viscosity in reducing post-print shrinkage in MatEx AM of rubbers. In that work, a liquid NBR polymer was used as the viscosity modifier for the NBR compounds under examination. In this study, we focused on evaluating the effectiveness of a plasticizer as a viscosity modifier for lowering compound viscosity and post-print shrinkage. Figure 5 shows the viscosity of the FKM compounds at the printing temperature of 100 °C and the percentage of shrinkage in printed parts after they were thermally cured.

Figure 5 clearly shows that there was a direct correlation between the compound viscosity and post-print shrinkage. The FKM compound without plasticizer showed almost three-fold more post-print shrinkage (17%, post cure) compared to the FKM compound with the plasticizer (6%, post cure). Such behavior can be attributed to the lower viscosity of the FKM compound with the added plasticizer. Due to the lower viscosity, the FKM compound with plasticizer experienced less induced stress during the extrusion process compared to the FKM compound without plasticizer. The reduced stress experienced by the polymer chains during extrusion for the plasticized FKM meant they were less stretched compared to the FKM without plasticizer [34,35,36]. Consequently, upon relaxation to their coiled configuration, FKM with plasticizer displayed a lower degree of post-print shrinkage in comparison to FKM without plasticizer. Overall, the lowered post-print shrinkage of the FKM compound with plasticizer validates the effectiveness of plasticizer as a viscosity modifier in lowering the post-print shrinkage in MatEx AM of fully compounded thermoset elastomers.

#### 3.1.3. Tensile Behavior and Interlayer Adhesion

**Figure 6:**
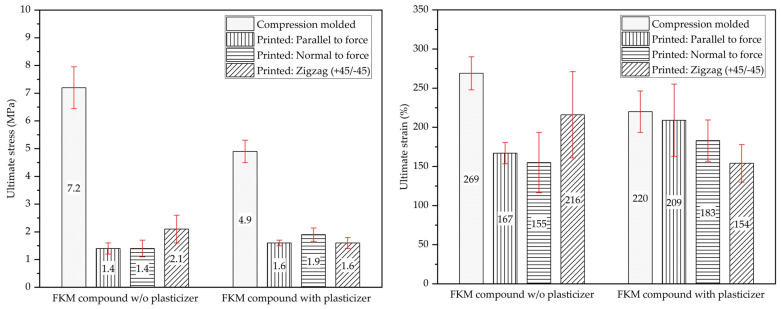
Tensile properties of compression-molded and printed samples of FKM compounds: (**left**) ultimate tensile stress and (**right**) ultimate tensile strain.

An additively manufactured part can be described as a chain of roads with multiple interfaces since it is fabricated by joining materials layer by layer. These layers join one another by the process of diffusion of polymeric chains and weld healing [37,38]. As a chain is as strong as its weakest link, an additively manufactured part is as strong as the strength of the adhesion between layers. In the literature, tensile testing is a well-established method of characterizing the strength of interlayer adhesion and overall integrity of printed parts [15]. In addition to studying the effectiveness of plasticizers on the viscosity and overall material printability, the effect on interlayer adhesion was also studied. As shown in Figure 3, three different types of printed tensile specimens, namely specimens with roads parallel to the direction of applied force, specimens with roads normal to the direction of applied force, and specimens with roads at +45°/−45° angle with the direction of applied force were evaluated and compared against the compression molded samples. In Figure 6, the tensile properties of both FKM compounds are presented and compared. Figure 6 also provides a side-by-side comparison of the tensile strength of a compression-molded and printed specimen. 

As can be seen in Figure 6, the compression-molded FKM compound with plasticizer is lower in ultimate tensile stress and strain by a noticeable amount when compared to the FKM compound without plasticizer. The effect of plasticizers on the mechanical properties is well known [21,22,23,24] and was expected. This behavior can be attributed to the increased free volume contributed by the plasticizer, making the rubber compound more flexible and less deformation-resistant [20,33]. 

**Figure 7:**
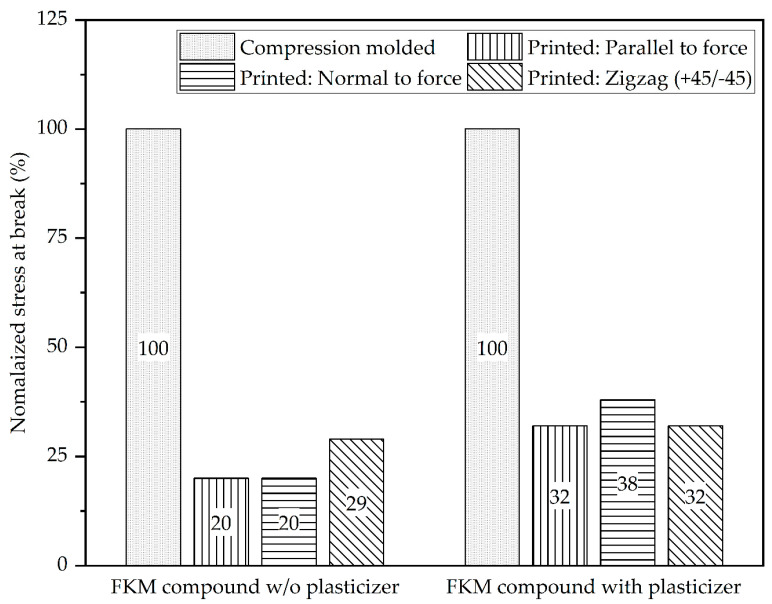
Normalized stress at break, an indication of interlayer adhesion of printed parts.

Similarly, one expects printed tensile specimens from FKM-2 (plasticized) to show lower tensile strength compared to unplasticized 3D-printed tensile specimens regardless of direction. Therefore, to compare the tensile properties of the printed parts, the effect of directionality on the properties, and the effect of plasticizer on interlayer adhesion, the normalized stress at the break of the printed samples was calculated and plotted in Figure 7. Normalized stress at break was defined as the ratio of the ultimate tensile stress of printed samples to compression-molded samples for each respective formulation. Normalized stress at break provides a simple measure to assess how much of the strength of the compression molded sample was retained in the printed samples and, thus, indicates the strength of the interlayer bonds of the FKM compounds. Although printed tensile specimens from both FKM compounds showed similar tensile properties, printed parts with the FKM-2 compound (with the plasticizer) showed higher normalized stress at break compared to the FKM-1 compound (without the plasticizer). Such behavior of the FKM compounds suggests that the printed parts with the FKM-2 compound retained more of their mechanical properties as measured by the compression-molded specimens. Such behavior can be attributed to the lower viscosity of the FKM compound with plasticizer, which may have facilitated better interdiffusion between adjacent roads and, thus, stronger interlayer adhesion [37,38]. In conclusion, there might be a trade-off when adding plasticizers to FKM compounds for AM applications. On the one hand, the plasticizer favorably affected the FKM compound’s overall printability by lowering the material viscosity. The lower viscosity helped to reduce the post-print shrinkage substantially and facilitated better interlayer adhesion between adjacent layers. However, adding the plasticizer to the FKM compound dropped the overall mechanical properties of the FKM compound.

### 3.2. Different Approaches for Additive Manufacturing of FKM Seals

FKM rubbers are widely employed in high-temperature and high-pressure sealing applications [1,2,3]. Therefore, AM technology for FKM rubbers holds substantial potential for the streamlined fabrication of these seals, which are traditionally manufactured via compression molding. Optimizing the AM technology for this specific purpose could enable rapid, cost-effective FKM seal prototyping, low-volume manufacturing, the fabrication of complex multiplanar seals, automated preform deposition for compression molding, and even in situ seal fabrication. To explore the feasibility of these applications, we investigated several conventional and unconventional AM methods. The following sections discuss these methods in detail.

#### 3.2.1. Traditional Additive Manufacturing of FKM Seals

In prior work, we demonstrated the feasibility of fabricating FKM- and FFKM-based seals and gaskets using the traditional FFF process by modifying a commercially available filament 3D printer and making filaments from FKM and FFKM compounds [18]. This work showed that the FFF process using FKM and FFKM filaments has a narrow process latitude, which caused reproducibility issues and shortcomings in meeting desired product specifications for very large and very small seals. In this work, the efforts were focused on increasing the process latitude to enable the printing of all types of seals ranging from large to medium and small seals. In the work, a large and complicated adapter seal was used due to its complicated design. 

**Figure 8:**
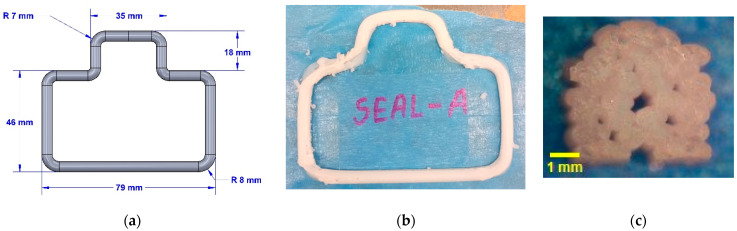
Conventional approach to additively manufacture a static seal from FKM rubber compound: (**a**) adapter seal geometry and dimensions, (**b**) printed adapter seal, and (**c**) cross-section of printed adapter seal.

The first attempt to additively manufacture FKM seals involved the traditional AM technique, in which the desired geometry was fabricated by layer-by-layer deposition of material. The desired geometry, in this case, was the adapter seal. The adapter seal is a static seal with a roughly 5 mm diameter circular cross-section. The seal geometry also had eight 90° sharp turns. Figure 8a provides the details of the seal geometry and its dimensions. Figure 8b shows the printed adapter seal at the end of the printing process but before the thermal cure of the FKM rubber. It can be seen from Figure 8b that the adapter seal fabricated using the conventional layer-by-layer AM method did not produce the desired shape. The most severe form of deformation occurred near the sharp turns. Post-print shrinkage of FKM rubber compounds resulting from the residual stress of extruded roads, as discussed in Section 3.1.1, can be attributed to these deformations. A closer examination of the cross-section of the printed adapter seal (Figure 8c) revealed the presence of a substantial number of voids. These voids, also known as pinholes, are typical characteristics of parts fabricated using the MatEx AM technique [39]. These voids are created when neighboring roads do not completely overlap with one another. Such voids usually weaken the printed part, but more importantly, in the case of sealing applications, these voids can lead to leakage and, eventually, failure of the seal [37]. Overall, the post-print deformation of the printed seal and the existence of characteristic pinholes in the cross-section made conventional layer-by-layer AM an unviable process for the fabrication of seals and gasket-like parts.

**Figure 9:**
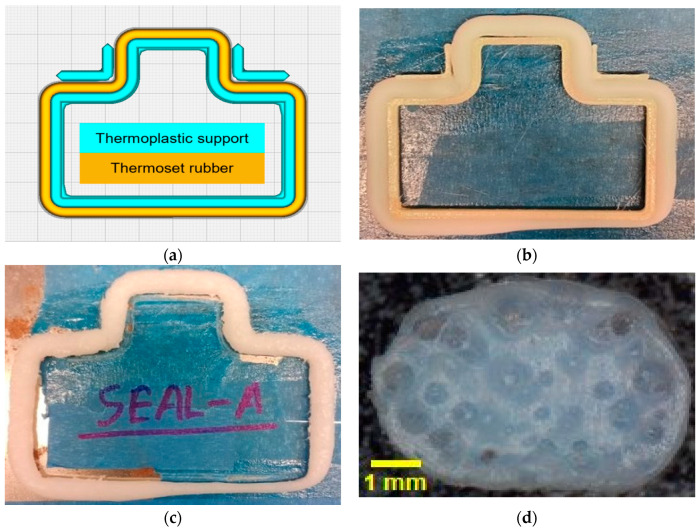
Traditional and direct additive manufacturing of an adapter seal with help of an enlarged nozzle and thermoplastic support structure: (**a**) 3D model of seal and thermoplastic support structure; (**b**) 3D-printed support structure and rubber seal; (**c**) 3D-printed rubber seal after thermal cure and removal of thermoplastic support structure; (**d**) cross-section of a 3D-printed FKM seal.

To prevent the post-print deformation of the printed adapter seal and eliminate the pinholes, thermoplastic support structures and a larger nozzle matching the cross-sectional diameter of the seal were employed. Drossel et al. [40] previously reported the use of silicone rubber, molding sand, and plaster as media for dimensional stabilization of 3D-printed parts from rubber compounds. However, in their work, the printed rubber geometry is fully embedded in the media. Contrary to such an approach, in our work, as depicted in Figure 9a, thermoplastic support structures were placed around the inside perimeter and at selected locations on the outside perimeter of the adapter seal. The fixture-like thermoplastic structures were printed first with commercial PLA filaments using the thermoplastic extruder attached to the ARME printhead. After the PLA support structures were printed, the adapter seal was printed using the FKM rubber compound. The adapter seal was printed using a nozzle with a larger outlet (4 mm nozzle outlet diameter) than conventional nozzles used in MatEx AM [15]. This approach was used to print the adapter seal with a layer height matching the adapter seal’s cross-sectional diameter. The reasoning behind such an unconventional approach was to eliminate the pinholes that were present when the seal was fabricated in multiple layers, which is the conventional approach of MatEx AM. Figure 9b shows the adapter seal printed in between the printed PLA support structures. It is evident from Figure 9b that the fixture-like PLA support structures prevented the extruded FKM rubber compound from deformation and allowed the acquisition of a substantially accurate printed part. Afterward, the adapter seal, along with the support structures, were placed inside a convection oven to be thermally cured at 125 °C. After completion of the curing process, the seal was taken out of the oven, and the support structures surrounding the seal were removed. Figure 9c shows the printed adapter seal after the cure and removal of support structures. The fully cured seal retained its shape even after the support structures were removed, which justified the effectiveness of using support structures. 

Additionally, the cross-section of the cured seal showed no presence of the pinholes, which was a result of using a large nozzle that matched the cross-section diameter of the seal. However, one drawback of employing a large nozzle matching the seal cross-section diameter was that there was only one seam where the nozzle completed the loop. This single seam was the weakest point of the seal and, as a result, was very prone to failure. Based on these results, a soft tooling approach to fabricating the adapter seal was employed to overcome the shortcomings of this approach and is discussed in detail in Section 3.2.2.

#### 3.2.2. Soft Tooling Approach for FKM Seal Fabrication

**Figure 10:**
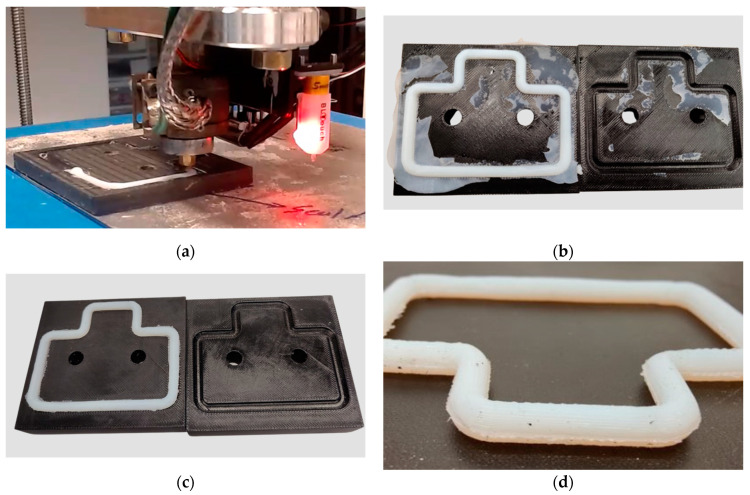
Soft tooling approach to fabricate seals: (**a**) deposition of uncured FKM rubber directly in the cavity of 3D-printed thermoplastic mold, (**b**) formed FKM seal with flash after completion of compression cycle, (**c**) FKM seal after removal of flash, (**d**) FKM seal after removal from 3D-printed thermoplastic mold/ soft tool.

Soft tooling is a manufacturing method that uses less durable and often more flexible materials to create molds, dies, or other production tools [41]. In this instance, the soft tool was a compression mold of the adapter seal additively manufactured with commercial acrylonitrile butadiene styrene (ABS) filament. In this approach, instead of printing on the print bed, the adapter seal was printed on the cavity of an ABS half mold, as shown in Figure 10a. The 4 mm nozzle matching the seal’s cross-sectional diameter was used for printing. The walls of the half-mold cavity performed the same role as the PLA support structures and prevented the extruded FKM rubber compound from shrinking and deforming. Once the seal printing process was completed, the other half of the mold was placed on top, and the setup was placed between a compression press. The mold was compressed with around 2 metric tons of force for 90 min at 100 °C. This method simulated the compression molding process and strengthened the seam situated at the point where the nozzle completed the loop. At the end of the compression cycle, the mold was removed from the press, the two halves of the mold were separated, and the flashed material was removed, as shown in Figure 10b,c, respectively. The adapter seal fabricated using this approach showed no post-print or post-cure deformation, as shown in Figure 10d. Upon closer inspection, no seamline was noticed, and the seal also retained its circular shape at its cross-section. Thus, using an additively manufactured soft tool, the adapter seal was fabricated through a hybrid process of AM and compression molding.

#### 3.2.3. Direct Printing of Molded-in-Place (MIP) Seals

**Figure 11:**
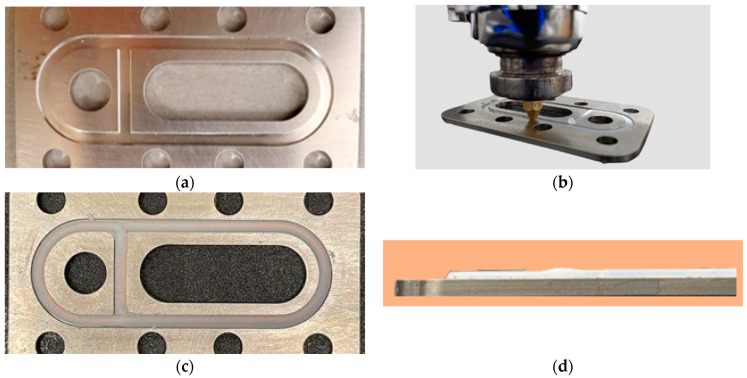
Additive manufacturing of bonded seal directly on seal housing: (**a**) metal seal housing; (**b**) deposition of FKM rubber compound directly inside of the seal housing; (**c**) printed seal after thermal cure (top view); (**d**) printed seal after thermal cure (right view).

As the name suggests, mold-in-place seals are formed by injecting the sealing material directly into the seal housing or groove. The sealing material creates a bond with the seal housing surface and, thus, forms a custom-fit seal. MIP seals offer superior sealing performance by reducing leak paths. They also offer enhanced environmental resistance as contaminants have no gaps to enter [42,43]. One of the significant advantages of MIP seals over conventional seals is their increased design flexibility, as they can conform to complex geometries. MIP seals find their application in numerous industries, such as automotive, aerospace, semiconductor, and consumer electronics [42,43]. MIP seals with FKM rubber as the sealing material are commonly used in the abovementioned industries where the seal needs to withstand high-pressure and high-temperature environments. Here, we have demonstrated the AM of an MIP seal directly into an aluminum metal housing (Figure 11a) using an FKM compound with a single extrusion layer, as shown in Figure 11b. After extruding the FKM compound into the seal housing, the seal housing acted similarly to the thermoplastic support structures reported in Section 3.2.1. They prevented the extruded FKM material from shrinking inwards and helped retain the material in place. Figure 11c shows a MIP seal directly printed into the seal housing (after thermal cure). This demonstration exhibits the feasibility of rubber AM technology in forming MIP seals. It also demonstrates the prospect of using the ARME print head to deposit rubber preform directly into the cavity of a compression mold, a process that is currently the predominant method of manufacturing in the rubber industry.

## 4. Conclusions

This study successfully demonstrated the potential of material extrusion additive manufacturing of functional FKM rubber components. Precise FKM deposition was demonstrated with the ARME extruder designed to print fully compounded thermoset elastomers.

The aim of this study was to evaluate the effectiveness of a plasticizer as a viscosity modifier and its effects on the 3D printability of an FKM rubber compound. In this regard, two FKM compounds, with and without plasticizer, were formulated and evaluated based on their viscosity, post-print shrinkage, tensile properties, and interlayer adhesion. The addition of a PFPE plasticizer reduced the compound viscosity and, thus, lowered the post-print shrinkage. The addition of the plasticizer also lowered the overall modulus of the cured compounds. The normalized stress at break for samples printed transverse to the stress direction was higher for the plasticized FKM compound as compared to the unplasticized compound indicating potentially better interlayer adhesion for the plasticized FKM. 

This study also evaluated the feasibility of rubber AM technology to fabricate FKM seals and gaskets for prototyping purposes. In this context, several conventional and unconventional AM approaches were evaluated. To better control the shape and dimensions of the printed parts, thermoplastic support structures were used. This approach was found to significantly improve the part quality. Last, a hybrid approach combining AM and soft tooling for compression molding was found to be the most promising fabrication method for FKM seals and gaskets.

These findings highlight the prospects of additively manufactured parts with tailored properties for various industrial applications. Future research directions could explore optimizing AM process parameters to further enhance the mechanical properties of printed FKM parts while maintaining printability. Additionally, investigating alternative plasticizers or formulations tailored for AM could lead to superior performance. Overall, this study paves the way for the development of additively manufactured FKM seals, gaskets, and other functional components with improved efficiency and design freedom. The information reported in this paper is also included in the two pending patents by Greene Tweed & Company [18,25].

## Figures and Tables

**Table 1:** Formulation of the two FKM compounds.

Compound/Recipe	Parts per Hundred Rubber (PHR)	
	FKM-1 (FKM w/o Plasticizer)	FKM-2 (FKM with Plasticizer)
Tecnoflon FKM (Tecnoflon VPL 45535-25 Mooney)	100	100
Filler and cure package for FKM	39	39
Plasticizer (Fomblin M60)	0	5

## Data Availability

Data are contained within the article.
